# Carcinoma of the Thyroid

**Published:** 1958-01

**Authors:** J. W. Bradbeer

**Affiliations:** Surgical Registrar, Cardiff Royal Infirmary Late S.H.O. in Surgery, Bristol Royal Infirmary


### CARCINOMA OF THE THYROID

### A Review of 100 Cases

BY

J. W. BRADBEER, M.B., F.R.C.S.

*Surgical Registrar, Cardiff Royal Infirmary*

*Late S.H.O. in Surgery, Bristol Royal Infirmary*

### INCIDENCE

Carcinoma of the thyroid is uncommon. In the South-West Region it accounts for only 0·69 per cent of all cancer, (excluding that of the skin). This compares with 0·4 per cent of a total of some 447,000 deaths from cancer (excluding the skin) notified in this country between 1951 and 1955.[1] In the Bristol Royal Infirmary since 1950, 1,096 patients with enlarged thyroids have been seen, and of these, 28 were malignant, an incidence of 2·5 per cent.

This paper reviews 100 cases of carcinoma of the thyroid occurring in the South-West Region of England. Half of these patients attended the Bristol Royal Infirmary in the first instance, the others were referred to the radiotherapy department at the Bristol General Hospital, having first been treated at other hospitals in the region. In this series there are 80 female and 20 male patients, a ratio of 4 to 1. Up to the age of 64, the ratio is 3·3 females to one male, whilst over 64, there were 31 females and only 5 males. The preponderance of females is peculiar, as the usual ratio is 2 females to 1 male. This anomaly is partly explained by the fact that only 5 of our patients were under the age of 40. In a recent report by Alhadeff *et al.*[2] from the Post-Graduate School at Hammersmith, one quarter of their patients were under 40 years of age, and in this age group the sex incidence was equal.

Nearly 40 per cent of cases occurred in the 55–64 age group, the youngest patient was 25, and the oldest 88 (Table 1).

TABLE I

AGE AND SEX INCIDENCE OF 100 CASES OF CARCINOMA OF THE THYROID

| Age | Males | Females | Total |
|---|---|---|---|
| 25–34 .. | 1 | 2 | 3 |
| 35–44 .. | 2 | 8 | 10 |
| 45–54 .. | 2 | 11 | 13 |
| 55–64 .. | 10 | 28 | 38 |
| 65–74 .. | 3 | 21 | 24 |
| 75–84 .. | 2 | 9 | 11 |
| 85–94 .. | 0 | 1 | 1 |
| Total .. | 20 | 80 | 100 |

### PRE-EXISTING GOITRES

It was widely believed that in most patients, thyroid carcinoma was preceded by adenomatous goitre. Cade[3] from 20 different papers on the subject, determined the average figure to be 71 per cent. In recent years however, clinicians have not supported this belief, and Alhadeff, Scott and Selwyn Taylor,[2] state that pre-existing goitre was present in only one-third of their cases.

If we define, as have these authors, a pre-existing goitre as a symptomless thyroid swelling present for more than 10 years, then only one-quarter of the patients in the present series fulfilled this criterion. In fact the length of history of a goitre in many patients was surprisingly short, one-third presented with a history of 3 months or less, and half with a history of up to 1 year.

This discrepancy in figures for the incidence of pre-existing goitre is probably due to 3 factors. First, the criterion for the length of time an enlarged thyroid must be present, to be classed as a pre-existing goitre, may vary from series to series. Secondly, in series from those areas where endemic goitre is common, there will be a higher percentage of cases with previous thyroid swelling. Lastly, Crile[4] has suggested that the slow rate of growth of the papillary type of cancer has given rise to the belief that the cancer arises in a pre-existing benign nodule. In this series, however, most of the cases of cancer arising in pre-existing goitre, have been of follicular type. The papillary type has appeared *de novo* with a history of 1–3 years. The anaplastic type has arisen in pre-existing goitres and *de novo* in equal proportions.

### MODES OF PRESENTATION

The modes of presentation of 78 of the best documented patients in this series have been analysed. Six clinical patterns are apparent (Table II).

TABLE II

AGE AND MODE OF PRESENTATION OF 78 CASES OF CARCINOMA OF THE THYROID

| Age | Lump in neck present less than $\frac{6}{12}$ | Lump in neck present $\frac{7}{12}$–3 years | Recent enlargement of pre-existing goitre | Difficulty in breathing | Thyrotoxicosis | Metastatic lesions | Total |
|---|---|---|---|---|---|---|---|
| 25–34 | 0 | 0 | 0 | 0 | 0 | 1 | 1 |
| 35–44 | 3 | 5 | 0 | 0 | 0 | 0 | 8 |
| 45–54 | 2 | 3 | 2 | 0 | 0 | 1 | 8 |
| 55–64 | 10 | 8 | 6 | 3 | 1 | 2 | 30 |
| 65–74 | 10 | 2 | 2 | 4 | 1 | 2 | 21 |
| 75–84 | 3 | 2 | 1 | 3 | 0 | 0 | 9 |
| 85–94 | 0 | 0 | 1 | 0 | 0 | 0 | 1 |
| Total | 28 | 20 | 12 | 10 | 2 | 6 | 78 |

1. *Lump present less than six months.* In this group, 28 patients, or over one-third of the total, mainly in the 55–64 age group, complained of a lump in the neck present for 6 months or less. In other words, they had a fairly rapidly enlarging thyroid swelling. In 5 patients with no other symptoms, 2 were diagnosed as having benign lesions, and in the other 3 a diagnosis of possible carcinoma was made. In most of the remainder, however, other symptoms occurred, the commonest being dyspnoea, followed by hoarseness, dysphagia, and occasionally pain in the distribution of the 2nd and 3rd cervical nerves. On examination the thyroid was enlarged unilaterally or bilaterally in equal numbers. Pathological examination revealed follicular carcinoma or anaplastic tumour in most cases. Eleven of the 28 cases died within 3 months, and only 8 survived for more than 2 years.

2. *Lump present for longer than six months.* Twenty patients, or about one-quarter of the total, presented with a lump in the neck which had been present for 1 to 3 years, that is, with a relatively slowly growing tumour. Patients in this group were a decade younger than those in the first group.

On examination 15 had unilateral enlargement of the gland, and 5 had enlargement of both lobes. Eight patients with unilateral enlargement are still alive, 5 of these had papillary carcinoma and all 5 have survived for more than 2 years after treatment. Of the 5 patients with bilateral enlargement 3 had anaplastic tumours and died within 6 months, the other 2 with papillary carcinoma are alive 3 and 4 years after treatment.

3. *Recent enlargement of pre-existing goitre.* In this group 12 patients, about one-sixth of the total mainly in the 55–64 age group, presented with recent enlargement of a pre-existing goitre.

The swelling was associated with dyspnoea, hoarseness and dysphagia in half the cases. In this group also, the diagnosis was not always definite, being classed as nodular goitre twice, and twice as "possible carcinoma". On examination, unilateral or bilateral goitre was found, in equal proportions. The thyroid was large, and hard on palpation. Pathological examination revealed follicular carcinoma in the majority. Six of these 12 patients are still alive up to 3 years after treatment.

4. *Difficulty in breathing.* Ten patients presented with a primary complaint of difficulty in breathing associated with thyroid swelling. One-half of these patients gave a history of long standing goitre, whilst in the remaining 5 there was a very short history of thyroid enlargement. On examination the glands were diffusely enlarged, hard, and in many cases, fixed. Breathlessness was so severe that emergency tracheostomy was performed. Six of these 10 patients were dead in 3 months, and 3 more within 6 months.

5. Two patients presented with symptoms and signs of thyrotoxicosis. The first, a female aged 67, underwent sub-total thyroidectomy. Papillary carcinoma was found on histological examination, and she is well two years after treatment. The second patient, a female aged 59, is free from recurrence or metastases four years after sub-total thyroidectomy. Follicular carcinoma was found on histological examination. Two other patients had a thyroidectomy carried out for thyrotoxicosis 9 and 10 years before presenting with carcinoma of the thyroid.

6. The final group of 6 patients presented with metastatic lesions. Five of these, all over 50 years and all females presented with osseous deposits.

The varied clinical picture in this group is shown in the following case histories.

CASE 1

**A female aged 54** presented with a 2-week history of swelling of the manubrium sterni, associated with pain, cough and breathlessness. One year previously a rib had been removed for a tumour, and thyroidectomy had been done ten years before. On examination there was a pulsatile swelling of the manubrium 4 cm. in diameter. Radio-active iodine uptake was high over the tumour, so a therapeutic dose was given. The mass completely disappeared within fourteen days. Histological examination of the rib tumour showed it to be a metatasis from carcinoma of the thyroid.

CASE 2

**A female aged 59** presented with aching pain in the back followed shortly by paraplegia. X-ray showed a secondary deposit in the tenth dorsal vertebra. The patient had had a swelling in the right side of her neck all her life, from which a biopsy was taken, which showed carcinoma of the thyroid. She died within four months.

CASE 3

**A female aged 60** consulted an orthopaedic surgeon about a pain in the leg, a metastatic deposit was found in the second lumbar vertebra on X-ray, and later one appeared in the cervical spine. A swelling in the neck had been present for years. Sub-total thyroidectomy was carried out and histological examination revealed carcinoma of the thyroid. The remainder of the gland was destroyed by radioactive iodine, and later, a tracer dose revealed functioning metastases. These metastases have been held in check by radioactive iodine for 5 years, the patient being still alive.

CASE 4

**A female aged 72** complained of a lump in the clavicle which had been gradually enlarging for 3 years.

Ten years previously, thyroidectomy had been carried out for nodular goitre. There was no uptake of radioactive iodine. On examination, the swelling, 5 cm. in diameter, showed expansile pulsation. It was excised, and shown to be a metastasis from thyroid carcinoma.

CASE 5

**A female aged 73** presented with a pathological fracture of the femur, and a large goitre. Biopsy of the neck swelling revealed carcinoma of the thyroid.

The only male patient in this group presented at the age of 25, with enlarged cervical lymph glands, which were at first diagnosed as being tuberculous.

### DIAGNOSIS

The diagnosis of carcinoma of the thyroid in its early stages has given rise to difficulty. Two patients, clinically diagnosed as having benign goitre, were found to have a malignant thyroid at operation. Nine patients, diagnosed as having a benign goitre clinically and at operation, were found to have carcinoma on histological examination. It is interesting that it is this group of patients with the wrong diagnosis that has the best prognosis (Table III).

*Single Nodules*. Five patients had an enlarged single nodule, thought to be innocent, until histological examination revealed a papillary carcinoma. The difficulty in diagnosis in these single nodules is well recognized. "Whether removal is urged because the nodule may become malignant, or because it is believed to be malignant from the start, most surgeons agree on immediate operation", (*Lancet*),[5] or to quote Lahey[6] "No discrete adenoma is too small to degenerate malignantly. The size of the thyroid tumour is no guarantee against the danger of its being malignant".

*Nodular Goitres*. At least 6 patients were diagnosed clinically as having benign nodular goitre. It is this type of case which presents the most difficult problem in diagnosis, for a history and clinical examination are no different from benign nodular goitre. Errors seem to be inevitable. One solution would appear to be to operate on all nodular goitres. This, however, is impracticable, as it has been estimated that one-quarter of a million thyroidectomies would have to be carried out in this country, (*Lancet*).[5] Moreover, in only one-quarter of the patients in this series did carcinoma arise in pre-existing goitre, so that this vast number of operations would have to be done to prevent only one-quarter of the cases of cancer of the thyroid. Again, even in endemic areas palpable nodular goitres represent only a small fraction of all the thyroids that actually contain nodules, (*Lancet*).[5] Crile[4] sums up the problem by stating that a woman would be better protected against a fatal cancer by removal of a normal breast or cervix, than by partial removal of a nodular goitre.

The diagnosis of carcinoma of the thyroid is not difficult in the later stages of the disease when patients present with a large hard mass in the neck, dyspnoea, dysphagia, recurrent laryngeal nerve paralysis, or pain due to involvement of the second and third cervical nerves. The disease at this stage may be mimicked, however, by other lesions, for example, infiltration by carcinoma of the oesophagus, bronchus, larynx, or breast, or by Riedel's thyroiditis, or Hashimoto's disease.

The following cases in which a diagnosis of carcinoma of the thyroid was made on clinical examination, are illustrative.

CASE 6

**A female patient aged 61** who had had a radical mastectomy 5 years previously, presented with a hard fixed swelling involving both lobes of the thyroid glands in both sides of the neck and left axilla. Biopsy showed spheroidal cell carcinoma of scirrhous type. At post-mortem neither lobe of the thyroid was recognizable. Trachea, larynx and oesophagus were embedded in dense malignant tissue, consistent with spread from carcinoma of the breast.

CASE 7

**A female patient aged 40** presented with increasing dysphagia and rapidly enlarging goitre. On examination there was a large swelling in the neck. At post-mortem a primary carcinoma of oesophagus was found with diffuse malignant infiltration of thyroid.

TABLE III

RESULTS OF TREATMENT OF 100 CASES OF CARCINOMA OF THE THYROID

| Number of cases | Clinical Diagnosis | Operative Diagnosis | Treatment | High voltage X-ray therapy | Number of patients alive | Shortest survival | Longest survival | Average survival |
|---|---|---|---|---|---|---|---|---|
| 5 | Single nodule, cyst or adenoma | Adenoma | Excision of adenoma | 3 | 5 | $\frac{6}{12}$ | $6\frac{2}{12}$ | $2\frac{7}{12}$ |
| 2 | Carcinoma | Carcinoma | Total thyroidectomy | 2 | 0 | $\frac{5}{12}$ | $4\frac{10}{12}$ | $2\frac{7}{12}$ |
| 1 | Nodular Goitre | Carcinoma | Attempted total thyroidectomy | 0 | 1 | — | — | $1\frac{3}{12}$ |
| 1 | Possible Carcinoma | Carcinoma | ,, ,, | 1 | 1 | — | — | 4 |
| 3 | Carcinoma | Carcinoma | ,, ,, | 3 | 1 | $\frac{1}{12}$ | $1\frac{7}{12}$ | $\frac{8}{12}$ |
| 4 | Nodular Goitre | Nodular Goitre | Sub-total thyroidectomy | 4 | 3 | $2\frac{2}{12}$ | 5 | $3\frac{7}{12}$ |
| 5 | Possible Carcinoma | Carcinoma | ,, ,, | 4 | 5 | $\frac{7}{12}$ | $2\frac{8}{12}$ | $2\frac{1}{12}$ |
| 6 | Carcinoma | Carcinoma | ,, ,, | 5 | 0 | $\frac{3}{12}$ | $\frac{6}{12}$ | $\frac{4}{12}$ |
| 8 | No record of clinical diagnosis | Carcinoma | ,, ,, | 8 | 4 | $\frac{9}{12}$ | $4\frac{7}{12}$ | $2\frac{4}{12}$ |
| 1 | Nodular Goitre | Carcinoma | Hemi-thyroidectomy | 1 | 1 | — | — | $4\frac{7}{12}$ |
| 3 | Carcinoma | Carcinoma | ,, ,, | 3 | 0 | $\frac{4}{12}$ | 1 | $\frac{8}{12}$ |
| 5 | No record of clinical diagnosis | Carcinoma | ,, ,, | 4 | 2 | $2\frac{8}{12}$ | $6\frac{3}{12}$ | $4\frac{2}{12}$ |
| 3 | Carcinoma | Carcinoma | Hemi-thyroidectomy and block dissection | 2 | 2 | $\frac{5}{12}$ | $6\frac{7}{12}$ | $2\frac{8}{12}$ |
| 4 | Carcinoma | Carcinoma | Partial removal for obstruction | 4 | 0 | $\frac{3}{12}$ | $1\frac{6}{12}$ | $\frac{9}{12}$ |
| 11 | Carcinoma | Carcinoma | Tracheostomy | 11 | 0 | 1 week | 3 | $\frac{5}{12}$ |
| 24 | Carcinoma | Carcinoma | — | 24 | 2 | 2 days | $3\frac{8}{12}$ | $\frac{9}{12}$ |
| 9 | Carcinoma | Carcinoma | Radio-active iodine | — | 3 | $\frac{3}{12}$ | $2\frac{11}{12}$ | $1\frac{6}{12}$ |
| 4 | Osseous metastases from thyroid carcinoma | | Radio-active iodine | 1 | 2 | $\frac{4}{12}$ | 5 | $2\frac{5}{12}$ |
| 1 | ,, | ,, | Excision of metastasis | — | 1 | — | — | $\frac{7}{12}$ |
| 100 | | | | 80 | 33 | | | |

CASE 8

A female patient aged 65 who had had a lump removed from the right side of the thyroid at 32, presented with fullness in the neck, dyspnoea, loss of voice, and pressure on the brachial plexus. Thyroidectomy was carried out. Histological examination of the thyroid revealed Hashimoto's disease.

The diagnosis is relatively easy when secondary deposits are the presenting feature, combined with the presence of a goitre or a history of recent thyroidectomy.

### RESULTS OF TREATMENT

The results of treatment are shown in Table III. If a definite diagnosis of carcinoma can be made, it is said that it is too late to carry out treatment which can offer any hope of permanent cure. It is often too late to carry out the only operation which can prevent the lethal effects of local recurrence, i.e., total or hemi-thyroidectomy. In fact, total thyroidectomy was performed only twice in this series. The first patient, a man of 49, with an anaplastic growth, survived for nearly 5 years, before he died from mediastinal metastases. The second patient, a female aged 69, had an anaplastic carcinoma, and died of cerebral metastases 3 months after operation.

Hemi-thyroidectomy was carried out in 12 cases, and was followed by irradiation in all except two. Five of these patients, all under 50, are still alive from 2–5 years after operation. Local recurrence causing death from respiratory obstruction occurred in 2 patients. There was evidence of spread outside the gland in one case, and invasion of blood vessels and lymphatics in the other at the time of operation. Hemi-thyroidectomy combined with block dissection of the neck, was carried out on three occasions.

When radical surgery was impossible, treatment has varied from attempted total thyroidectomy, sub-total thyroidectomy, through partial removal for obstruction, to exploration and biopsy only, all with or without the addition of irradiation. In 5 cases a diagnosis of benign nodule or cyst was made pre-operatively, and removal of the nodule only was undertaken, (despite Crile's warning that if a thyroid nodule is considered dangerous enough to warrant removal it should be regarded as malignant, and therefore hemi-thyroidectomy should be performed rather than "shelling out," or local removal).[5] In fact these 5 patients are alive from 6 months to 6 years after treatment. Papillary carcinoma was found in each case.

Radiotherapy was the sole method of treatment in 24 patients, who had advanced inoperable cancer, 11 of them having glandular or pulmonary metastases at presentation. It is not surprising, therefore, that 10 were dead in 3 months. Radiotherapy alone had not been given to any operable cases in this series.

Thirty-five patients received tracer doses of radio-active iodine. In only 2 was there any evidence of uptake by the gland. One was a female aged 67, with a papillary type of growth, sub-total thyroidectomy was followed by a therapeutic dose of radio-active iodine. The patient became myoedematous, confirming the ablation of the gland. Tracer doses 2 years later failed to show functioning metastases. The patient is still alive, two years after treatment. The second patient, a female aged 58, with a follicular carcinoma, that had been partially removed, was treated by radio-active iodine. The growth recurred however, and the patient died 2 years later with widespread metastases. Radio-active iodine was given to 7 other patients after thyroidectomy, or exploration and biopsy, without affecting the course of the disease to much extent.

If the treatment of the primary growth with radio-active iodine in this series, has been disappointing, it has been remarkably successful in two cases with osseous metastases. These are the two cases referred to above, who presented with secondary deposits, one in the manubrium sterni (Case 1), the other in the lumbar and cervical spine (Case 3).

Correlation of the histological types of carcinoma of the thyroid to the results of treatment has not been attempted. The reason for this is that histological examination of all the tumours has not been carried out and classified by any one pathologist.

From the table it can be seen that 16 out of 17 patients in whom a clinical diagnosis of benign enlargement of the thyroid, or "possible carcinoma" was made, are alive from 6 months to 6 years after treatment. Only 8 out of 70 patients in whom a definite clinical diagnosis of carcinoma was made are still alive.

### METASTASES

Metastatic deposits in bone which were the presenting feature in 5 patients, appeared later in a further seven. They appeared in the clavicle, manubrium sterni, skull, ribs, vertebrae, pelvis and femur. The primary lesion was follicular or anaplastic. In this series no osseous deposits from a papillary type of primary growth were found.

Enlarged cervical lymph nodes were felt in 20 cases at presentation and appeared later in further 7. The type of growth in these cases was mainly anaplastic or papillary.

Metastases, mainly from anaplastic growths, were found in the lungs in 14 cases. Deposits in the liver and brain were also encountered.

In summary, papillary carcinoma metastasised mainly to regional lymph glands, and soft tissue organs, but never to bone, follicular tumours metastasised mainly to bone and soft tissues. Anaplastic growths spread to all structures.

### MODE OF DEATH

The commonest mode of death was respiratory obstruction from recurrent or uncontrolled growth. The mechanism appears to be either narrowing of the trachea, causing death by asphyxiation, or perforation, associated with haemorrhage, bronchopneumonia following due to aspirated blood and growth. The next most frequent causes were cachexia associated with multiple metastases, followed by death from pulmonary deposits.

### SUMMARY

1. One hundred cases of Thyroid cancer have been reviewed.
2. The ratio of females to males was 4 to 1.
3. In only 25 per cent of patients, was there pre-existing goitre.
4. Early diagnosis proved difficult, and therefore radical surgery was rare.
5. Occasionally there was difficulty in diagnosing the apparently late case.
6. The commonest mode of death is respiratory obstruction.
7. The 3 year survival rate under the age of 50 was 40 per cent, it was only 20 per cent over the age of 50.
8. The five year survival rate of treated cases from 1945–1950 in the South-West Region is 26 per cent.
9. The prognosis is bad if a definite diagnosis of carcinoma can be made on clinical examination, conversely the prognosis is relatively good if a mistaken diagnosis of benign goitre, or "possible carcinoma" is made.

I wish to acknowledge the help of Professor R. Milnes-Walker, at whose suggestion this review was carried out, the help of Dr. R. C. Tudway, who made available the notes of the patients treated at the Bristol General Hospital, and the permission of those surgeons in the South-West Region under whose care the patients were admitted.

### REFERENCES

[1] *Registrar-General's Statistical Review of England and Wales* (1951–1955). London: H.M.S.O.

[2] Alhadeff, R., Scott, F., and Selwyn Taylor (1956), *Brit. J. Surg.*, **43**, 617.

[3] Cade, S. (1949), *Malignant Disease and its Treatment by Radium.* 2nd. ed., Vol. II. 380. Bristol: Wright.

[5] *Lancet* (1956). *Annotation*, **271**, 665.

[6] Lahey, F. H. (1938). *Surg. Clinics of North America*, **18**, 587.

